# *Toxoplasma gondii* seropositivity associated to peri-urban living places in pregnant women in a rural area of Buenos Aires province, Argentina

**DOI:** 10.1016/j.parepi.2019.e00121

**Published:** 2019-10-09

**Authors:** Elías M. Rivera, Silvina N. Lavayén, Paola Sánchez, Carlos M.A. Martins, Etelvina Gómez, Jorge P. Rodríguez, Marcela E. Arias, Andrea P. Silva, Sergio O. Angel

**Affiliations:** a Laboratorio de Parasitología Molecular, INTECH, Consejo Nacional de Investigaciones Científicas y Técnicas (CONICET)/Universidad Nacional General San Martin (UNSAM), Int. Marino Km8.2, Chascomús, Provincia de Buenos Aires, CP7130, Argentina; b Laboratorio de Bacteriología, Instituto Nacional de Epidemiología Dr. Juan H. Jara-ANLIS Dr. Carlos G. Malbrán, Mar Del Plata, Provincia de Buenos Aires, Argentina; c Hospital Municipal San Vicente de Paul, Chascomús, Provincia de Buenos Aires, Argentina; d Secretaría de Salud, Municipalidad de Chascomús, Provincia de Buenos Aires, Argentina

**Keywords:** Toxoplasmosis, Seroprevalence, Rural, Poverty

## Abstract

Infection with *Toxoplasma gondii* is very common in humans throughout the world, the intake of raw or undercooked meat with tissue cysts and fruits, vegetables and water contaminated with parasite oocysts being the main routes of infection. Here, we analyzed the seroprevalence of *anti*-*T. gondii* antibodies in pregnant females (age 13–44 years; n = 920) between April 2014 and December 2017 from Chascomús (Argentina), a city immersed in a rural area. Altogether 320 tested positive for immunoglobulin G antibodies, yielding an overall seroprevalence of 34.8% (CI 95%: 31.7–37.9). No association was observed between seropositivity and age. In addition, by using the QGIS 3.2.1 software we analyzed the geographical distribution of 769 (83.6%) pregnant females in two main areas of the city: Urban (n = 157) and Peri-urban (n = 612) with a seroprevalence of 26.8% (CI 95%: 19.8–33.7) and 36.4% (CI 95%: 32.6–40.3) respectively, and this difference was statistically significant (p = 0.023). Furthermore, we assessed through a questionnaire survey, between April 2016 to December 2017, possible risk factors such as activity (urban and rural), home water supply, animal husbandry, presence of cats as pets, gardening and consumption of meat and its derivatives (pork, sheep meat and sausages) and their frequencies (consumption per week), not finding significant association with seropositivity. Significant differences was found when the seroprevalence was analyzed between the urban and peri-urban neighborhoods of the city of Chascomús. The higher seroprevalence in peri-urban neighborhoods could be due to an unfavorable socioeconomic situation and/or to undeveloped peri-urban environments, which is a risk factor that should be taken into account when planning the health care of pregnant females.

## 1 Introduction

*Toxoplasma gondii* is an obligate intracellular parasite which belongs to the Phylum Apicomplexa, with felines as the definitive hosts and all the warm-blooded animals, including humans, as intermediate (Jiang et al., 2018). This parasite is the causative agent of toxoplasmosis, a zoonotic disease that infects one third of the human population and it is considered one of the most important infections produced by food (Khan and Khan, 2018) (Bojar and Szymańska, 2010). Acute infection can be asymptomatic or cause non-specific symptoms that include fever, lymphadenopathy and myalgia, imitating other infectious diseases (Theel and Pritt, 2016). However, *T. gondii* infection presents serious implications in particular in immunocompromised patients and newborns in the case of congenital transmission, where tachyzoites can cross the placenta and infect the fetus, with clinical manifestations ranging from spontaneous abortion, intrauterine growth retardation, hydrocephalus, neurological alterations, retinochoroiditis, cardiovascular anomalies, to an asymptomatic newborn that manifest symptoms, such as eye damage, at some period of his life (Dard et al., 2017) (Avelar et al., 2018).

The main route of infection include the consumption of raw or undercooked meat (Araújo et al., 2018) and its derivatives that have tissue cysts, direct contact with oocysts present in the soil through gardening and the consumption of contaminated fruits and poorly washed vegetables (Awoke et al., 2015) (Kaufer et al., 2017) or by consuming untreated well water (Da Silva et al., 2015; Krueger et al., 2014). Currently, the seroprevalence of *T. gondii* is between 1 and 90% but these values vary according to the region, climatic differences, socioeconomic conditions, food and hygiene habits and the susceptibility of the host (Fallahi et al., 2018) (Dard et al., 2016). All these factors can explain why the prevalence is extremely variable between different countries and in different regions within the same country (Da Silva et al., 2015). There is further evidence that the risk of contracting *T. gondii* infection increases when socio-economic conditions are unfavorable such as poverty or low quality of life (Awoke et al., 2015; Kaufer et al., 2017; Da Silva et al., 2015; Fallahi et al., 2018; Dard et al., 2016). However, in some cases it was observed that higher socio–economic status coincides with higher consumption of raw meat correlating with high seroprevalence (Jones et al., 2018). Interestingly, living in a rural area or working on farm tasks was also associated with a higher seroprevalence of *anti*-*T. gondii* antibodies (Wilking et al., 2016; Alvarado-Esquivel et al., 2013; Inagaki et al., 2014). Although there are several studies that compare the levels of seroprevalence between rural and urban regions, there are few studies that address the incidence of toxoplasmosis between urban and peri-urban/suburban regions. A study conducted in Aracaju, Brazil, found higher levels of seroprevalence in women from peri-urban neighborhoods, in this case coinciding with an unfavorable economic situation (Inagaki et al., 2014).

Argentina is a country with a strong rural component. However, the main studies to detect *T. gondii* antibodies were carried out with populations associated with cities. Seroprevalences of toxoplasmic infection of 21.2% were observed in blood donors in Buenos Aires D. C. (CABA), although in recent years a decrease has been observed (Kaufer et al., 2017). In pregnant women the seroprevalence observed in CABA was 18.33% (Carral et al., 2013). In order to estimate the seroprevalence of the *T. gondii* infection in a rural area, we investigated the levels of seroprevalence in pregnant women of Chascomús, a region of strong association with rural activity and given that there are important differences in the urban development within the city of Chascomús, we analyzed whether there was association with seroprevalence in less developed areas (peri-urban region) as was observed by others. In addition, we also analyzed possible risk factors that could be associated with *T. gondii* infection to determine possible routes of infection.

## 2 Materials and methods

### 2.1 Area of study

The study was conducted in Chascomús city ((35° 34′ 30″ S, 58° 0′ 32″ W) located within the Province of Buenos Aires, 123 km from (CABA). The total population of the city is 42,277 inhabitants of which 21,570 are women (Ministerio de Economía de, 2011). It has an area of 4163.19 km^2^ with an altitude of 10 m above sea level, with low soils typical of the region, especially suitable for extensive livestock farming. The climate is humid temperate to sub-humid, with an average annual temperature of 16 °C, being the average for the summer of 23 °C and in the winter of 9 °C (Montero, 2009). Only 76.9% of households have treated water, 55.9% with sewer services and 44.5% natural gas. The main economic activity lies in the manufacturing industry (40.1%) and 8.4% of the total is represented by agriculture, livestock and forestry, where the bovine rearing represents 1.14% of the total of the province; the rest is represented by public administration, transport and communications, commerce, construction and tourism. Regarding health coverage, Chascomús has a public hospital, Hospital Municipal San Vicente de Paul, six public health centers dependent of this hospital and a private health service (Caviglione, 2011).

### 2.2 Study population

A retrospective study was performed in the programmatic area of the Hospital Municipal San Vicente de Paul. The inclusion criteria were to consider all pregnant females who carried out their antenatal cares in the Hospital Municipal San Vicente de Paul, according to the norms of antenatal control of the Ministry of Health of the Argentine Nation and of the Province of Buenos Aires about prevention of congenital toxoplasmosis and gave birth in this hospital between April 2014 and December 2017. The *T. gondii* serology includes the detection of immunoglobulin G (IgG) and immunoglobulin M (IgM) antibodies. IgG test was performed by using the Indirect Hemagglutination Assay (IHA) test (HAI; Wiener Lab. Group, Argentina). Positive individuals for IgG antibodies were tested for IgM antibodies by using IgM- Enzyme-linked Fluorescent Immunoassay (ELFIA; Biomerieux, France). Both tests are commercially available and were performed according to the manufacturer's specifications and are part of routine assays of San Vicente de Paul's Hospital Clinical Diagnostic Unit. Of note, IgG-IHA test is suitable for mass screening in epidemiological studies (Liu et al., 2015) (Tenter et al., 2000).

### 2.3 Risk factors

A questionnaire survey was completed by pregnant females from April 2016 to December 2017, to analyze possible risk factors associated with *T. gondii* infection. The risk factors analyzed were: Activity (urban, urban/rural or rural), water supply at home (potable from public water company or well water), animal husbandry, presence of cats as pets, gardening and consumption of meat and their frequencies (pork, sheep meat, sausages).

### 2.4 Geographical distribution

The geographical coordinates of individual's address of pregnant females that participated in this study were obtained to determine their distribution in the city. Chascomús city was divided in developed urban (all public services, fully inhabited) and undeveloped peri-urban (lack of some public services, partially inhabited, presence of empty lots) area modified from Tauber (1993) (Tauber, 1993), by using the QGIS 3.2.1 software.

### 2.5 Statistical analysis

All data were analyzed using *Epi Info* 7.2.2.6 and Epidat 3.1. The prevalence ratio (PR) and the 95% confidence intervals (CI) were calculated to assess the possible association of variables.

The chi-square test was used to evaluate significant association between the values of prevalence of *anti*-*T. gondii* antibodies observed in urban and peri-urban areas. A *p*-value < 0.05 was considered statistically significant.

### 2.6 Ethical considerations

This study was approved by the ethics committee (FEMEBA Nota Nro 502, 23sep 2014) and the individuals signed an informed consent for the use of the data for research purposes. According to the local laws, minor of the age range of this study can sign the consent themselves.

## 3 Results and discussion

### 3.1 Prevalence of *anti*-T. gondii antibodies and risk factors

In this period of study, 1182 individuals gave birth at the Hospital Municipal San Vicente de Paul; 86 individuals gave birth more than once in this period and from them we included the results of the last serology performed (seroconversion was not observed in seronegative individuals) and 176 individuals did not have present serology data. Thus, data from 920 pregnant females were collected (77.8%), with an age range between 13 and 44 years (mean age of 25.10 ± 6.1 years old). Of these 920 individuals, 320 were positive for IgG antibodies (Table 1). None of these 320 individuals had positive serology for IgM antibodies, which shows a profile of chronic infection.

**Table 1:** Age-associated prevalence of *anti*-*T. gondii* antibodies in pregnant women from Chascomús, from April 2014 and December 2017.

Variable	No. Participants	IgG + (%)	PR (95% CI)
Age (year)			
<19	169	54 (31.9)	1
20–34	671	231 (34.4)	1.08 (0.84–1.37)
>34	80	35 (43.8)	1.37 (0.98–1.91)

**PR**: prevalence ratio. **95% CI**: 95% confidence interval.

The overall prevalence of *anti*-*T. gondii* antibodies was 34.8% (320/920) (CI 95%: 31.7–37.9). This prevalence value is higher than the 18.33% observed in a study conducted on 12,035 pregnant women from Hospital Alemán from CABA (Carral et al., 2013). Since the study carried out by the Hospital Alemán was based on the Sabin Feldman *Dye Test,* a more sensitive test than the one used in the present work, this could not account for the difference observed. A possible explanation is that Chascomús is a rural area which was demonstrated to be a risk factor for *T. gondii* infection as observed by others (Rostami et al., 2016) (Wilking et al., 2016). In the city of Chascomús only part of the individuals are linked to rural activities, independent of the area where they live, and it could be observed that the seroprevalence among people with farm or rural activities in comparison with individuals that never had any relationship with rural activities was not significantly different (Table 2). This indicates that other risk factors could affect the high infection rate observed in the Chascomús population.

**Table 2:** Prevalence of *anti*-*T. gondii* antibodies in pregnant women from Chascomús, by the investigated variables, from April 2016 to December 2017.

Risk Factor	No. Participants		Seropositive (%)	PR (95% CI)
**Activity**				
Urban	261	82 (31.4)		1
Urban/Rural	57	17 (29.8)		0.95 (0.61–1.47)
Rural	8	4 (0.5)		1.59 (0.78–3.26)
**Water supply**				
Well water	92	32 (34.8)		1
Treated	195	55 (28.2)		1.23 (0.86–1.76)
**Animal husbandry**				
Yes	82	29 (35.4)		1
No	223	67 (30.0)		1.18 (0.83–1.68)
**Presence of cat**				
Yes	84	23 (27.4)		1
No	230	77 (33.5)		0.82 (0.55–1.21)
**Gardening**				
Yes	34	11 (32.4)		1
No	287	92 (32.1)		1.01 (0.60–1.69)

**PR**: prevalence ratio. **95% CI**: 95% confidence interval.

The analysis of other risk factors such as water supply at home (potable or well water), animal husbandry, presence of cats as pets, gardening (Table 2), did not show a significant association with *T. gondii* infection. This is in agreement with several studies that did not show an association between pets or gardening with seropositivity with this parasite (Fallahi et al., 2018) (Alvarado-Esquivel et al., 2013). In this study no association was found with the water supply. Of note, homes with well water, which has high saline content, usually drink commercial bottled water, which may be an explanation of the observed result. Age was not a risk factor in this study, but the pregnant females with an age >34 years had the highest seroprevalence (43.2%).

Argentina is known as a meat consumer country, specially from bovine animals (Gallup, 2005). To notice, the consumption of porcine and ovine meat has increased in recent times, which are known as possible sources of *T. gondii* infection (Oliveira et al., 2018) (Dubey, 2009). Therefore, we investigated different meat types as potential risk factor. None of the individuals included in this study were vegetarian. The consumption of meat and their frequencies (Table 3) did not show significant association with *T. gondii* seropositivity. In general, in Chascomús, as in other rural areas, all meats are cooked well done, maybe explaining the lack of differences among the type of meats.

**Table 3:** Prevalence of *anti*-*T. gondii* antibodies in pregnant women from Chascomus, by reported meat consumption, from April 2016 to December 2017.

Risk Factor	No. Participants	Seropositive (%)	PR (95% CI)
**Pork**			
No	108	30 (27.8)	1
Once and less than once a week	187	58 (31.0)	1.12 (0.77–1.62)
More than once	32	14 (43.8)	1.58 (0.96–2.59)
**Sheep**			
No	139	41 (29.5)	1
Once and less than once a week	160	50 (31.2)	1.06 (0.75–1.50)
More than once	29	11 (37.9)	1.29 (0.76–2.19)
**Sausages**			
No	57	22 (38.6)	1
Once and less than once a week	211	55 (26.1)	0.68 (0.45–1.01)
More than once	59	24 (40.7)	1.05 (0.67–1.65)

**PR**: prevalence ratio. **95% CI**: 95% confidence interval.

### 3.2 Distribution

**Fig. 1:**
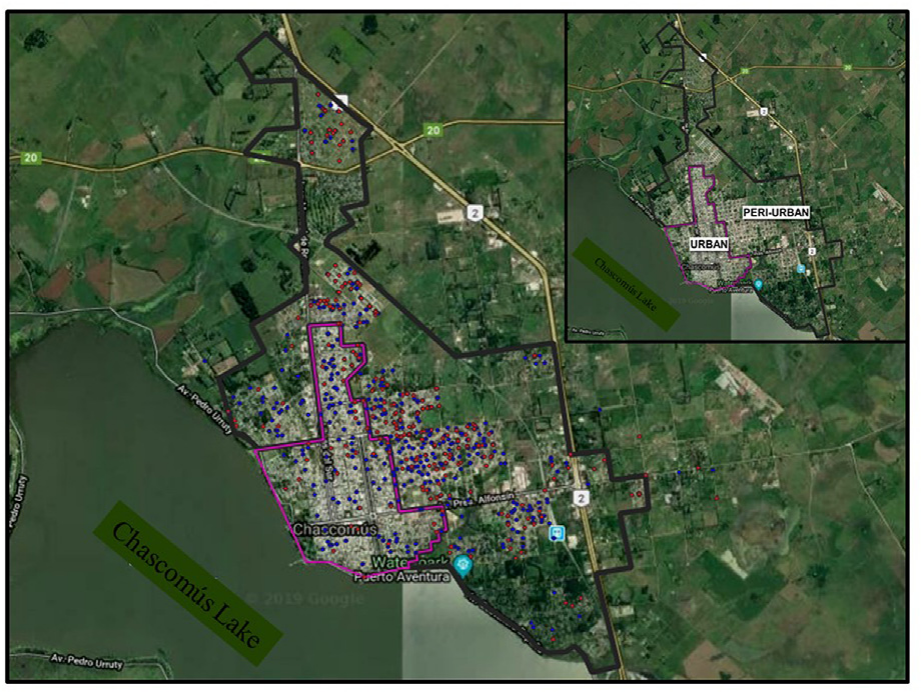
Distribution of pregnant women in Urban and Peri-urban area. Red: Individual IgG positive for *T. gondii* antibodies. Blue: Individual IgG negative for *T. gondii* antibodies. Corner: Map of the City of Chascomús with the two main area: URBAN and PERI-URBAN. Map data: ©2019 Google.

Of the 920 individuals with serology data, geographical coordinates of 769 (83.6%) households were obtained taking into account the address provided in their clinical history. By using the QGIS 3.2.1 software these individuals could be located in the city and their distribution determined in two main areas: urban and peri-urban (Fig. 1). Thus, 157 had declared their home in urban areas (mean age of 25.81 ± 6.01 years old) and 612 in peri-urban areas (mean age of 25.18 ± 6.15 years old) (Fig. 1). The prevalence of *anti*-*T. gondii* antibodies in urban and peri-urban areas was 26.8% (CI 95%: 19.8–33.7) and 36.4% (CI 95%: 32.6–40.3) respectively, and this difference was statistically significant (p = 0.023). A large population is concentrated in the urban area in respect to the total population. Here, there is a very low availability of uninhabited land and is the most developed area since it has a greater coverage in basic needs, such as access to treated water, sewer and gas services. In the peri-urban area, as we move away from the downtown area towards the periphery, this development is impoverished as there are areas with no access to gas and sewer services and only a minority has access to treated water. At the same time, there is more uninhabited land and green spaces that could be visualized at the map as green patches between homes (Fig. 1). Although additional studies are necessary to evaluate the reasons for the differences found, two possible causes could be cited based on the literature: differences in socioeconomic levels between urban/peri-urban area and or the fact that domestic cats in the peri-urban area have more access to rodent predation activities than in the urban region as observed by others (Inagaki et al., 2014; Afonso et al., 2013). More studies are needed to unravel whether these are the causes of the differences in seroprevalence we observed in Chascomús.

## 4 Conclusions

In the present study, high frequency of anti-*Toxoplasma gondii* antibodies (IgG class) was found in pregnant females, being higher than that observed in the federal district (CABA). This indicates a higher rate of infection that is tempting to associate with rural activity within Chascomús; however, no differences were observed in the present work. Moreover, there were no associations between *T. gondii* infection and the different food sources analyzed or the presence of cat as a pet and with the water supply in Chascomús. Interestingly, a higher prevalence was associated with peri-urban neighborhoods compared to the more urbanized neighborhoods in the center, suggesting undeveloped peri-urban environments as a risk factor that should be taken into account in the health care of pregnant females. Given that there are few studies comparing peri-urban with urban neighborhoods of the same city, the results obtained here could encourage similar studies in other regions. Finally, we encourage generating guidelines to prevent *T. gondii* transmission in these neighborhoods, and monitoring the situation.

## Conflicts of interest

The authors hereby declare that there is no conflict of interest in the study.

## References

[bib1] Afonso E., Germain E., Poulle M.L., Ruette S., Devillard S., Say L. (2013). Environmental determinants of spatial and temporal variations in the transmission of *Toxoplasma gondii* in its definitive hosts. Int J Parasitol Parasites Wildl.

[bib2] Alvarado-Esquivel C., Campillo-Ruiz F., Liesenfeld O. (2013).

[bib3] Araújo A.C., Villela M.M., Sena-Lopez A., da Rosa Farias N.A., Jorge de Farias L.M., Farias da Costa Avila L. (2018). Seroprevalence of *Toxoplasma gondii* and *Toxocara canis* in a human rural population of southern Rio Grande do Sul. Rev. Inst. Med. Trop. Sao Paulo.

[bib4] Avelar J.B., Gontijo da Silva M., Hanstter Hallison A.R., Ribeiro Storchilo H., Naves do Amaral W., Rodrigues Xavier I. (2018). Epidemiological factors associated with *Toxoplasma gondii* infection in postpartum women treated in the public healthcare system of goiânia, state of Goiás, Brazil. Rev. Soc. Bras. Med. Trop..

[bib5] Awoke K., Nibret E., Munshea A. (2015). Sero-prevalence and associated risk factors of *Toxoplasma gondii* infection among pregnant women attending antenatal care at Felege Hiwot Referral Hospital, northwest Ethiopia. Asian Pac J Trop Med.

[bib6] Bojar I., Szymańska J. (2010). Environmental exposure of pregnant women to infection with *Toxoplasma gondii* - state of the art. Ann. Agric. Environ. Med..

[bib7] Carral L., Kaufer F., Olejnik P., Freuler C., Durlach R. (2013). Prevención de la Toxoplasmosis congénita en un hospital de Buenos Aires. Medicina B Aires.

[bib8] Caviglione J. (2011). Plan estratégico de turismo sustentable en el partido de Chascomús, Ministerio Económia y de la Producción de La Nación. https://www.mininterior.gov.ar/planificacion/pdf/planes-loc/BUENOSAIRES/Desarrollo-Local-en-Chascomus-Propuestas-del-Plan.pdf.

[bib9] Da Silva M.G., Vinaud M.C., De Castro A.M. (2015). Prevalence of toxoplasmosis in pregnant women and vertical transmission of *Toxoplasma gondii* in patients from basic units of health from Gurupi, Tocantins, Brazil, from 2012 to 2014. PLoS One.

[bib10] Dard C., Fricker-Hidalgo H., Brenier-Pinchart M.P., Pelloux H. (2016). Relevance of and new developments in serology for toxoplasmosis. Trends Parasitol..

[bib11] Dard C., Chemla C., Fricker-Hidalgo H., Brenier-Pinchart M.P., Baret M., Mzabi A. (2017). Late diagnosis of congenital toxoplasmosis based on serological follow-up: a case report. Parasitology.

[bib12] Dubey J.P. (2009). Toxoplasmosis in sheep—the last 20 years. Vet. Parasitol..

[bib13] Fallahi S., Rostami A., Nourollahpour Shiadeh M., Behniafar H., Paktinat S. (2018). An updated literature review on maternal-fetal and reproductive disorders of *Toxoplasma gondii* infection. J Gynecol Obstet Hum Reprod.

[bib14] Gallup T. (2005). El consumo de carne vacuna en la argentina.

[bib15] Inagaki A.D., Cardoso N.P., Lopes R.J., Alves J.A., Mesquita J.R., de Araujo K.C., Katagiri S. (2014). Análise espacial da prevalência de toxoplasmose em gestantes de Aracaju, Sergipe, Brasil. Rev. Bras. Ginecol. Obstet..

[bib16] Jiang R.L., Ma L.H., Ma Z.R., Hou G., Zhao Q., Wu X. (2018). Seroprevalence and associated risk factors of *Toxoplasma gondii* among Manchu pregnant women in northeastern China. Microb. Pathog..

[bib17] Jones J.L., Kruszon-Moran D., Scott Elder, Rivera H.N., Press C., Montoya Jose G. (2018). *Toxoplasma gondii* infection in the United States, 2011–2014. Am Soc Trop Med Hyg.

[bib18] Kaufer F.J., Carral L.A., Messina M.T., Schneider M.V. (2017). Prevalencia de anticuerpos anti *Toxoplasma gondii* en hemodonantes en la ciudad de buenos aires, desde 1967 a 2017. Medicina B Aires.

[bib29] Krueger W.S., Hilborn E.D., Converse R.R., Wade T.J. (2014). Drinking water source and human *Toxoplasma gondii* infection in the United States: a cross-sectional analysis of NHANES data. *BMC Public Health*.

[bib19] Khan K., Khan W. (2018). Congenital toxoplasmosis: an overview of the neurological and ocular manifestations. Parasitol. Int..

[bib20] Liu Q., Wang Z.D., Huang S.Y., Zhu X.Q. (2015). Diagnosis of toxoplasmosis and typing of *Toxoplasma gondii*. Parasit vectors.

[bib21] Ministerio de Economía de la Provincia de Buenos Aires (2011). Censo 2010 Provincia de Buenos Aires Resultados Definitivos por Partido.

[bib22] Montero J.C. (2009). Contribución al desarrollo local y regional de Chascomús a través de la actividad turística.

[bib23] Oliveira G.C., de Souza Almeida H.M., Sartori R.S., Rossi G.A.M., de Oliveira L.G., Langoni H. (2018). Prevalence of *Toxoplasma gondii* infections in swine of non-tecnified rearing farms of the northeastern region of the state of São Paulo, Brazil and associated risk factors. Parasite Epidemiol Control.

[bib24] Rostami A., Seyyedtabaei S.J., Aghamolaie S., Behniafar H., Lasjerdi Z., Abdolrasouli A. (2016). Seroprevalence and risk factors associated with *Toxoplasma gondii* infection among rural communities in northern Iran. Rev. Inst. Med. Trop. Sao Paulo.

[bib25] Tauber F. (1993). Chascomús: pautas para una estrategia de desarrollo, Munic. Chascomús.

[bib26] Tenter A.M., Heckeroth A.R., Weiss L.M. (2000). *Toxoplasma gondii*: from animals to humans. Int. J. Parasitol..

[bib27] Theel E.S., Pritt B.S. (2016). Microbiol. Spectr..

[bib28] Wilking H., Thamm M., Stark K., Aebischer T., Seeber F. (2016). Prevalence, incidence estimations, and risk factors of *Toxoplasma gondii* infection in Germany: a representative, cross-sectional, serological study. Sci. Rep..

